# Protein Kinase C Promotes Notch1 Cleavage Through Both Ligand-Dependent and Hyaluronic Acid–ADAM10-Dependent Mechanisms

**DOI:** 10.3390/cells15151349

**Published:** 2026-07-27

**Authors:** Xin Tang, Yunfei Mu, Hongjun Shi

**Affiliations:** 1 College of Life Sciences, Zhejiang University, Hangzhou 310058, China; tangxin@westlake.edu.cn; 2 Key Laboratory of Growth Regulation and Translational Research of Zhejiang Province, School of Life Sciences, Westlake University, Hangzhou 310024, China; muyunfei@westlake.edu.cn; 3 Westlake Laboratory of Life Sciences and Biomedicine, Hangzhou 310024, China; 4 Institute of Basic Medical Sciences, Westlake Institute for Advanced Study, Hangzhou 310024, China

**Keywords:** protein kinase C, Notch signaling, NICD, hyaluronic acid, HAS2, ADAM10, Jagged1, endothelial cells

## Abstract

**Highlights:**

**What are the main findings?**

- PKC activates Notch signaling in endothelial cells through two parallel mechanisms: transcriptional upregulation of Jag1 and hyaluronic acid-dependent maintenance of ADAM10 protein levels required for Notch proteolytic cleavage.
- Direct phosphorylation of Notch1 at S1791 is dispensable for PKC-induced Notch activation, indicating that PKC acts through upstream, indirect mechanisms.

**What are the implications of the main findings?**

- The PKC–Has2–hyaluronic acid signaling axis provides a mechanistic explanation for the phenotypic similarity between Has2-deficient and Notch1-deficient mouse embryos.
- Pharmacological targeting of PKC downstream effectors may offer therapeutic strategies for atherosclerotic lesions and congenital valve malformations.

**Abstract:**

Notch1 signaling is essential for endothelial cell fate determination and vascular development. While PKC has been implicated as an upstream activator of Notch1 signaling, the molecular mechanisms by which PKC promotes Notch1 proteolytic cleavage remain poorly defined. Here, using murine endothelial MS1 cells and mouse aortic endothelial cells (MAECs), we identify two cooperative pathways through which PKC drives generation of the Notch1 intracellular domain (NICD). First, PKC activation increases mRNA and protein levels of the Notch1 ligands Jag1 and Dll4. A NICD-Dll4 positive feedback loop further amplifies Notch signaling. Second, PKC upregulates hyaluronic acid synthase 2 (HAS2) mRNA and protein expression, promoting hyaluronan (HA) synthesis. Various genetic (HAS2 knockdown, CD44 knockdown, Hyal2 overexpression) and pharmacological (4-methylumbelliferone, 4-MU) treatments confirmed that HA is required for PKC to activate Notch1. Inhibition of HA production or signaling is associated with downregulation of ADAM10 on the cell surface. Direct phosphorylation of Notch1 at S1791 is dispensable for this effect. Together, these findings reveal a dual pathway model in which PKC coordinates ligand upregulation and HA signaling to promote Notch1 cleavage, identifying the extracellular matrix as a previously unrecognized regulator of Notch1 signal strength in endothelial cells.

## 1. Introduction

Notch signaling plays an essential role in the development of the cardiovascular system [1,2]. In the past decades, a considerable amount of work has been done to delineate the primary cues that establish the pattern of Notch activation during early mouse embryogenesis, the period when Notch is required for vasculogenesis and arterial fate specification [3,4]. These studies conclusively identified the Notch ligand Dll4 as the primary inducer of Notch activation during early phases of vascular development [5,6,7]. The regulation of Notch activation in the developing heart is more complex. In the developing endocardium (the endothelial lining of the heart lumen), Notch activation is highest in the endocardial cells overlying the atrioventricular canal (AVC) and proximal outflow tract (OFT)—regions fated to form valves and septa [8,9]. This pattern of activation does not appear to depend on Dll4, as Dll4 expression in the AVC and OFT endocardium at this stage remains very low compared to that in the vascular endothelium [8,10]. Our recent work demonstrated that high shear stress activates Notch in the AVC and OFT endocardium in a protein kinase C (PKC)-dependent manner [10]. However, the signaling cascade linking PKC to Notch activation remains unknown.

The Notch signaling pathway operates via a juxtacrine mechanism and requires ligand binding from adjacent cells [11,12]. Notch receptors undergo S1 cleavage by furin in the Golgi, forming a heterodimer that is transported to the plasma membrane. Upon binding to DSL ligands on neighboring cells, mechanical force generated by ligand endocytosis exposes the S2 site on the extracellular side of the receptor, allowing the transmembrane protease ADAM10/17 to cleave [11,13,14,15]. The remaining membrane-tethered fragment is then cut at the S3 site by γ-secretase, releasing the Notch intracellular domain (NICD). NICD translocates to the nucleus, where it binds CSL transcription factors, recruits Mastermind (MAML), and activates target gene expression [11,16]. Consequently, PKC may promote Notch signaling by regulating any component of the pathway, including ligand expression.

While the mechanism by which PKC activates Notch in the endocardium remains elusive, insights from other biological systems may inform our understanding of this signaling axis. Notably, Ligand-independent, PKC-dependent Notch activation has been reported in activated T cells following TCR stimulation both in vivo and in vitro [17,18]. During T cell activation, PKC induces Coronin 1A phosphorylation, facilitating the formation of large actin-based lamellipodia and the recruitment of ADAM10 to the T cell/antigen-presenting cell interface, leading to Notch activation [18]. PKC has also been shown to phosphorylate Notch1 at S1791, promoting its processing in late endosomes/lysosomes and thereby enhancing nuclear translocation [19]. However, whether these mechanisms operate in endothelial cells is still unclear. In this study, we analyzed the factors that promote Notch activation in cultured endothelial cells. We found that PKC activates Notch1 through a mechanism involving both ligand and hyaluronic acid.

## 2. Materials and Methods

### 2.1. Cell Culture and Treatment

MS1 (MILE SVEN 1) cells were obtained from Cell Bank, Chinese Academy of Sciences (Cat# GNM20). Mouse aortic endothelial cells (MAECs) were obtained from stemrecell (Cat# CL0200, Fenghui Biology, Shanghai, China). The mouse endothelial cell line was cultured under standard conditions. Chemical reagents included Phorbol 12-myristate 13-acetate (PMA, 10 ng/mL 3 h, MedChemExpress (MCE), HY-18739), Staurosporine (1 μM 3 h, MCE, HY-15141), Cycloheximide (50 ng/mL 3 h, MCE, HY-12320), Actinomycin D (50 ng/mL 3 h, MCE, HY-17559), DAPT (1 μM 3 h, MCE, HY-13027), 4-Methylumbelliferone (1 μM 12 h, MCE, HY-N0187), MG-132 (10 μM 6 h, MCE, HY-13259). All compounds were dissolved in DMSO and diluted in culture medium.

### 2.2. siRNA and Plasmid Transfection

Small interfering RNAs (siRNAs) targeting Prkce (Protein kinase C epsilon, PKCε), Has2 and Cd44 were synthesized by Sangon Biotech (Shanghai, China). The sequences were as follows: siPrkce: Sense: 5′-CGUCACUUCGAGGACUGGAUU-3′, Antisense: 5′-AAUCCAGUCCUCGAAGUGACG-3′. siHas2: Sense: 5′-UUACGGAAAUGUUUGCAAdTdT-3′, Antisense: 5′-AUUGCAAACAUUUCCGUdTdT-3′ [20]. siCD44: Sense: 5′-UUCCUUCGAUGGACCGGUU-3′, Antisense: 5′-AACCGGUCCAUCGAAGGAA-3′ [21]. MS1 cells were transfected with siRNAs using Lipofectamine RNAiMAX reagent (Thermo Fisher Scientific, Waltham, MA, USA), and with Hyal2 plasmids (P51510, Miaoling Bio, Wuhan, China) using Lipofectamine 3000 Transfection Reagent (Thermo Fisher Scientific, Waltham, MA, USA) according to the manufacturer’s instructions.

### 2.3. Western Blotting

Cells were lysed in 1× SDS-PAGE Sample Loading Buffer (P0015B, Beyotime, Shanghai, China) supplemented with protease and phosphatase inhibitors (P002, NCM biotech, Suzhou, China). Proteins were separated via SDS-PAGE and transferred to PVDF membranes. Membranes were blocked in 5% milk and incubated overnight at 4 °C with the following primary antibodies: Phospho-(Ser) PKC Substrate (#2261S, Cell Signaling Technology, Danvers, MA, USA, CST), Cleaved Notch1 (Val1744) (#4147S, CST), PKC epsilon (#2683S, CST), Notch1 (#3608S, CST), Jagged1 (#70109S, CST), Has2 (PA5-115388, Thermo Fisher Scientific), Hyal2 (PA5-115387, Thermo Fisher Scientific), Cd44 (ab157107, Abcam, Cambridge, UK), Adam10 (ab124695, Abcam), Dll4 (AF1389, R&D Systems), ATPase (AF1864, Beyotime, Shanghai, China), β-Tubulin (A12289, Abclonal, Wuhan, China), Gapdh (AC002, ABclonal). Secondary antibodies conjugated to HRP were applied, and signals were detected using enhanced chemiluminescence.

### 2.4. Activation of NICD in 293T Cells

Notch1-WT, Notch1-SA S1791A, and Notch1-SE S1791E plasmids were constructed according to a published study [19]. All Notch constructs have their C-terminus truncated to differentiate from the endogenous Notch1. Plasmids were transfected into 293T cells using Lipo8000™ Transfection Reagent (Beyotime). NICD activation in 293T cells was performed as follows: cells were treated with PMA (10 ng/mL) for 30 min, washed with PBS, and then treated with EDTA (0.025 mM) for 10 min.

### 2.5. RNA Extraction and Quantitative Real-Time PCR (qPCR)

Total RNA was isolated using TRIzol reagent (15596018, Invitrogen, Carlsbad, CA, USA) following the manufacturer’s protocol. Complementary DNA (cDNA) was synthesized, and qPCR was performed to quantify gene expression. The primer sequences used were: Dll4 Forward: 5′–ggaaccttctcactcaacatcc–3′, Reverse:5′–ctcgtctgttcgccaaatct–3′), Jag1 Forward: 5′tctctgacccctgccataac–3′, Reverse: 5′–ttgaatccattcaccagatcc–3′) [22], Has2: Forward: 5′-TGCTTGACCCTGCCTCATC-3′, Reverse: 5′-CCCATGAATTCCTGATTGTACCA-3′ [23]. *Gapdh*: Forward: 5′-ACCACAGTCCATGCCATCAC-3′, Reverse: 5′-TCCACCACCCTGTTGCTGTA-3′ [24]. Relative mRNA levels were normalized to *Gapdh* using the 2^−∆∆Ct^ method.

### 2.6. Bulk RNA Sequencing and Bioinformatics Analysis

Total RNA was isolated using TRIzol reagent (15596018, Invitrogen, Carlsbad, CA, USA). The RNA samples were then shipped to Shanghai Majorbio Bio-Pharm Technology Co., Ltd. (Shanghai, China) for library construction and sequencing. Sequencing was performed on an Illumina Novaseq 6000 platform (Illumina, San Diego, CA, USA). Raw sequencing data were first subjected to quality assessment using FastQC (v0.11.9), and potential contaminations were detected using FastQ_Screen (v0.15.3). Raw reads were trimmed and filtered using fastp (v0.23.4). Clean reads were aligned to the mouse reference genome GRCm38 using HISAT2 (v2.2.1). Alignment results were converted to BAM format using samtools (v1.14). Gene-level read counting was performed using the featureCounts function from the R package Rsubread (v2.26.0) based on the Ensembl v102 (GRCm38) GTF annotation file. Differential expression analysis was conducted using the R package DESeq2 (v1.42.1), with |log2 fold change| ≥ 1 and *p* ≤ 0.05 as the screening thresholds. Volcano plots were generated using the R package ggplot2 (v3.5.2) to visualize differentially expressed genes.

### 2.7. Immunofluorescence (IF) and Hyaluronic Acid Detection

MS1 cells were fixed and blocked with 10% normal donkey serum, followed by incubation with primary antibodies. For HA staining, cells were sequentially incubated with biotinylated hyaluronic acid binding protein (385911, Sigma, St. Louis, MO, USA), Cy3 streptavidin, and anti-CD31 antibody (#3528, CST, Danvers, MA, USA,). Nuclei were counterstained with DAPI (Beyotime, C1002). Images were acquired on a Zeiss LSM 800 microscope (Carl Zeiss, Oberkochen, Germany).

### 2.8. Cell Surface Biotinylation

MS1 cells were washed three times with ice-cold PBS and incubated with 0.5 mg/mL Sulfo-NHS-SS-Biotin (Cat#A8005-100, Apexbio, Houston, TX, USA) in ice-cold PBS for 30 min at 4 °C with gentle rocking. The biotinylation reaction was quenched by washing three times with ice-cold 100 mM glycine in PBS. Cells were lysed in NETN buffer (20 mM Tris-HCl pH 8.0, 100 mM NaCl, 1 mM EDTA, 0.5% NP-40) supplemented with protease inhibitor cocktail for 30 min on ice. Lysates were clarified by centrifugation at 12,000× *g* for 15 min at 4 °C. Equal amounts of protein were incubated with Streptavidin Sepharose beads (Cat#17-5113-01, GE Healthcare, Chicago, IL, USA) overnight at 4 °C with rotation. Beads were washed four times with NETN buffer, and bound proteins were eluted by boiling in 2× SDS sample buffer for 10 min. Eluates were subjected to SDS-PAGE and immunoblotted for ADAM10. Na^+^/K^+^-ATPase served as a plasma membrane loading control.

### 2.9. Statistical Analysis

All data are expressed as the mean ± SEM of at least three independent experiments (specific n values are indicated in figure legends) and were tested for normality and homogeneity of variance. Statistical significance was determined by two-way ANOVA followed by Tukey’s post hoc test, one-way ANOVA followed by Tukey’s post hoc test, or unpaired Student’s *t*-test. All analyses were performed with GraphPad Prism 10 (GraphPad Software, San Diego, CA, USA). Exact *p* values are reported in the figures; differences were considered significant at *p* < 0.05.

## 3. Results

### 3.1. Notch1 Activation by PKC in Endothelial Cells

**Figure 1:**
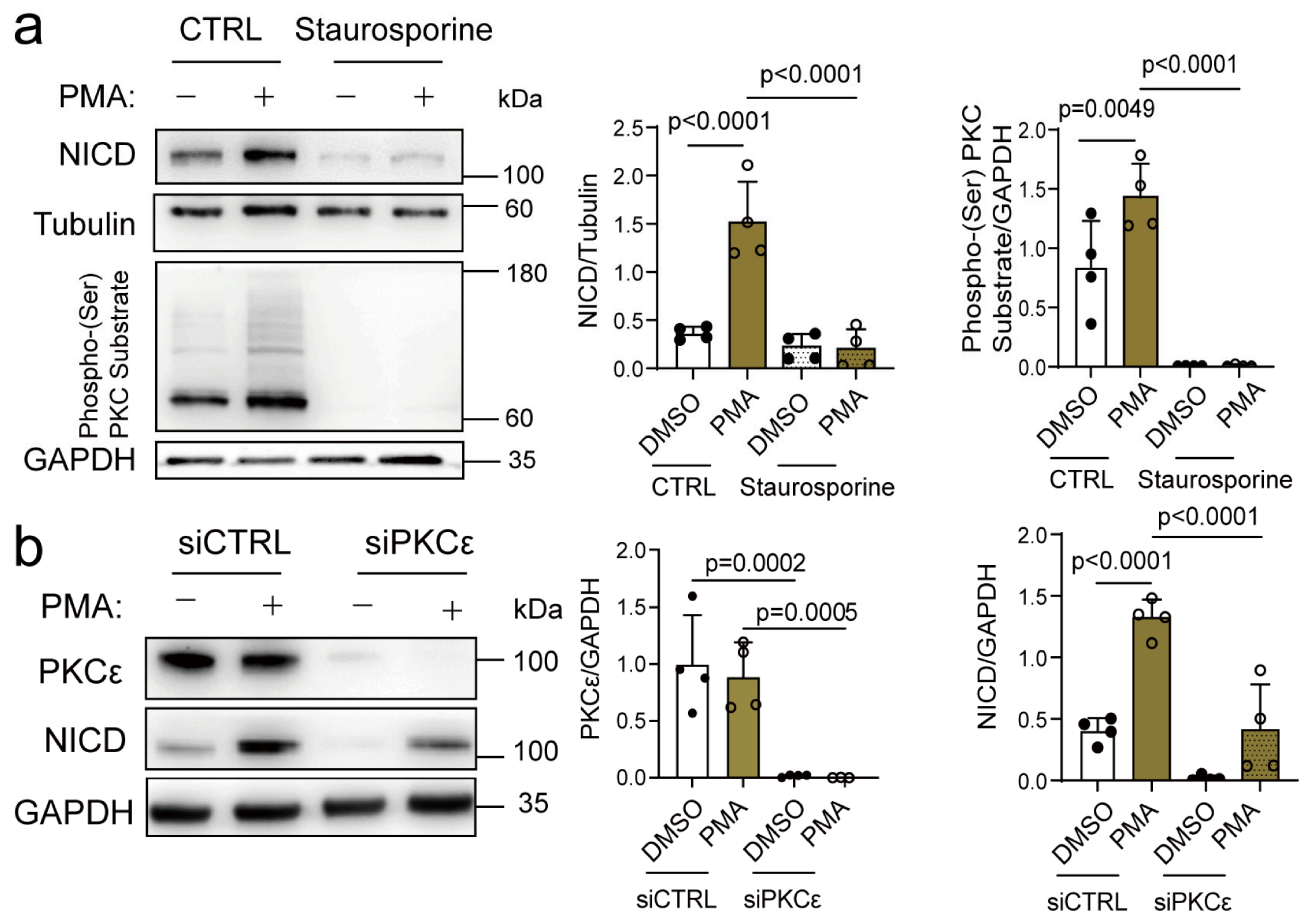
PMA-induced NICD generation is dependent on PKC activity. MS1 cells were treated with PMA (10 ng/mL, 3 h) in the presence or absence of (**a**) the PKC inhibitor Staurosporine (1 μM, co-treatment) or (**b**) PKCε-specific siRNA (siPKCε, 48 h pre-transfection). NICD, phospho-(Ser) PKC substrate (p-PKCsub), and PKCε protein levels were analysed by Western blot. Representative blots are shown. The protein levels were normalised to α-Tubulin. The graphs display the means ± SEM (n = 4). Exact *p* values are indicated in the graphs; statistical analysis was performed using two-way ANOVA followed by Tukey’s post hoc test.

Previous studies have demonstrated that PKC is critical for Notch activation and valve morphogenesis in the developing mouse endocardium. To investigate the underlying molecular mechanism, we treated cultured endothelial MS1 cells with PMA, a well-established PKC activator. As shown in Figure 1a, the Notch1 cleavage product, Notch1 intracellular domain (NICD), was significantly upregulated after 3 h of PMA stimulation, whereas treatment with the PKC inhibitor staurosporine abolished both basal NICD levels and the stimulatory effect of PMA. Changes in the levels of phosphorylated (Ser) PKC substrates confirmed the efficacy of PKC activation and inhibition. The inhibitory effect of staurosporine was recapitulated by silencing Prkce, which also significantly inhibited Notch activation under both basal and stimulated conditions (Figure 1b). These findings were independently reproduced in mouse aortic endothelial cells (MAECs), where both staurosporine treatment and Prkce knockdown similarly blocked PMA-induced NICD generation (Figure S1).

### 3.2. Activation of NICD by PKC Is Independent of S1791 Phosphorylation

**Figure 2:**
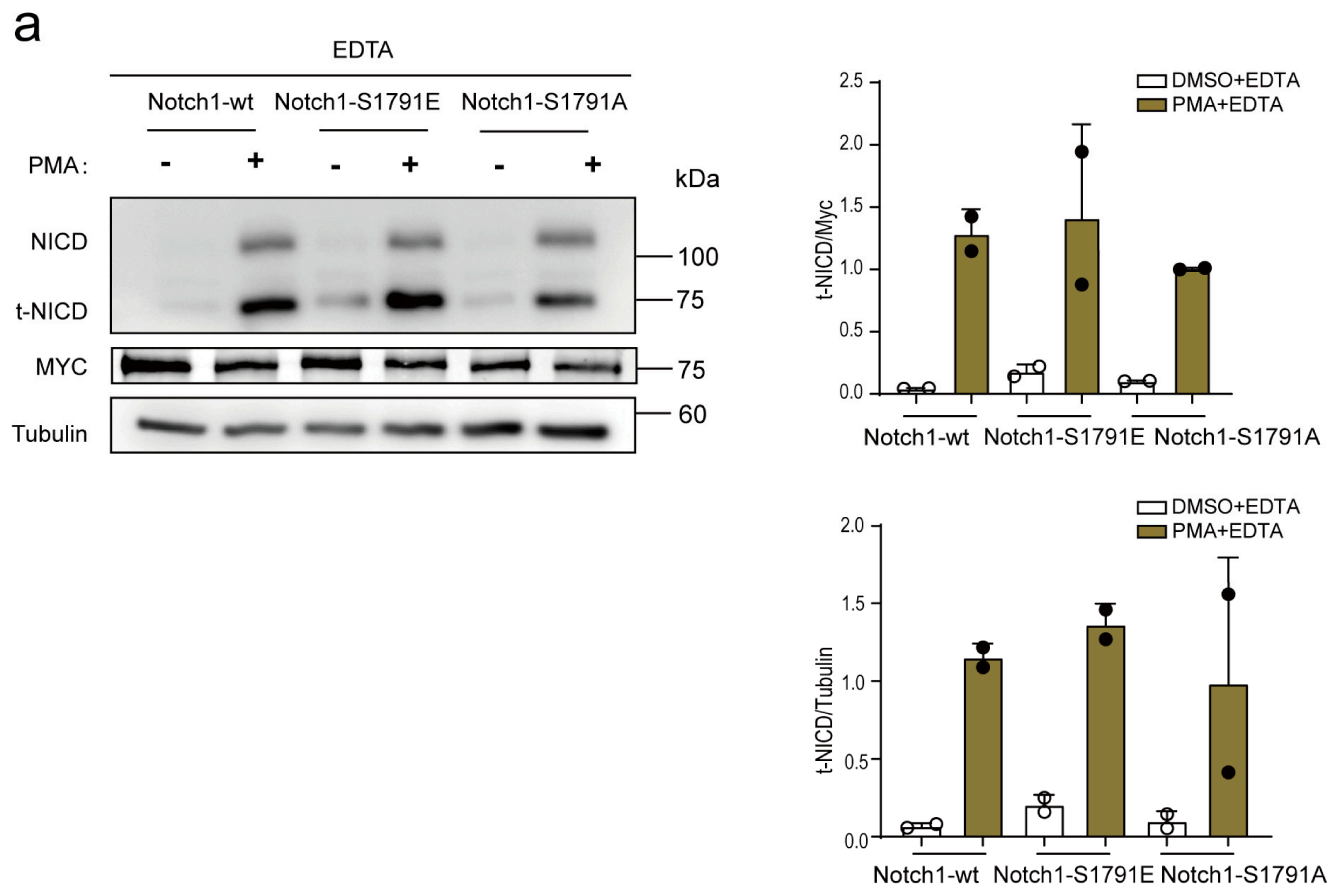
Notch1 S1791 phosphorylation is not required for PKC-mediated NICD generation. (**a**) 293T cells were transfected with wild-type Notch1 (Notch1-WT), phosphomimetic mutant (Notch1-S1791E), or phosphorylation-deficient mutant (Notch1-S1791A), and subsequently treated with PMA (10 ng/mL, 30 min) and EDTA (0.025 mM, 10 min) to stimulate Notch1 cleavage. Truncated NICD (tNICD) derived from the transfected constructs was detected by Western blot. α-Tubulin was used as a loading control.

To verify whether PMA mediates NICD activation through phosphorylation of the Notch1 S1791 site, we examined the effect of PMA on NICD production in 293T cells. Although 293T cells lack functional Notch1 ligands, they express the Notch1 receptor and are therefore suited for studying Notch1 signaling activation. Wildtype Notch1 (Notch1-WT) as well as its phosphorylation-deficient (Notch1-SA S1791A) and phosphomimetic (Notch1-SE S1791E) mutants were generated following the method described by Sjöqvist et al. [19]. These plasmids were transfected into 293T cells, followed by sequential treatment with PMA and EDTA. Western blot results demonstrated that all three constructs respond to PMA to produce NICD at comparable levels (Figure 2a). Collectively, these findings indicate that PMA effectively promotes NICD production regardless of the mutational status of the S1791 site, suggesting that this residue is not involved in PKC-induced Notch1 activation in endothelial cells.

### 3.3. PKC Promotes NICD Activation by Upregulating the Expression of Notch Ligands

**Figure 3:**
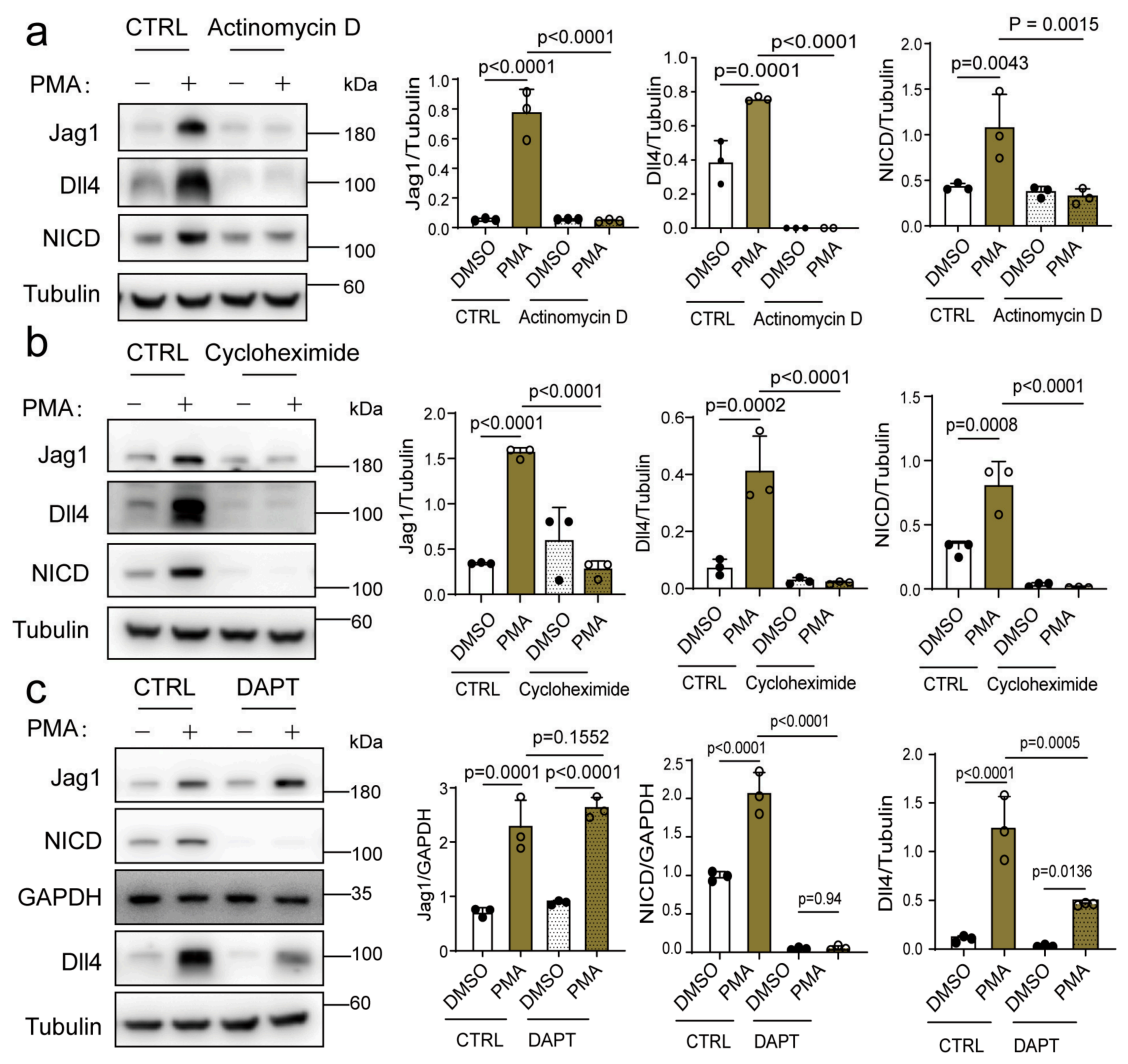
PKC promotes NICD generation through upregulation of Notch1 ligands. (**a**–**c**) Western blot analysis and densitometric quantification of Jag1, Dll4, and NICD protein levels in MS1 cells treated with PMA (10 ng/mL, 3 h) in the presence or absence of (**a**) the transcription inhibitor Actinomycin D (ActD, 50 ng/mL), (**b**) the translation inhibitor Cycloheximide (CHX, 50 ng/mL), or (**c**) the γ-secretase inhibitor DAPT (10 μM). α-Tubulin and GAPDH were used as loading controls. Data are presented as mean ± SEM (n = 3 independent experiments). Exact *p* values are indicated in the graphs; statistical analysis was performed using two-way ANOVA with Tukey’s post hoc test.

PKC phosphorylates a wide range of target proteins and may perturb gene transcription programs. We first analyzed the effect of PKC activation on Notch1 ligand expression. PKC activation significantly upregulated the two main ligands in MS1 cells, Dll4 and Jag1, at both protein and mRNA levels in a time-dependent manner; Jag1 and Dll4 expression progressively increased from 1 to 3 h of PMA treatment, paralleled by a corresponding increase in NICD generation (Figure S2). Treatment with actinomycin D and cycloheximide inhibited the upregulation of these ligands and subsequent Notch activation, suggesting that newly synthesized ligands may be responsible for Notch activation (Figure 3a,b). To examine whether Jag1 or Dll4 is the dominant responder, we treated cells with the γ-secretase inhibitor DAPT. DAPT completely blocked NICD production and significantly inhibited Dll4 expression under both basal and stimulated conditions. In contrast, Jag1 protein levels were unaffected by DAPT (Figure 3c). These results suggest that PKC primarily upregulates Jag1 expression, which in turn activates Notch, whereas Dll4 upregulation occurs downstream of Notch signaling and may contribute to Notch activation via a positive feedback mechanism. Furthermore, pharmacological inhibition of ADAM10 with GI254023X completely abolished PMA-induced NICD generation (Figure S3), confirming that ADAM10 protease activity is required for PKC-mediated Notch1 S2 cleavage.

### 3.4. PKC Activation Upregulates Has2 mRNA Levels and Hyaluronic Acid Deposition

**Figure 4:**
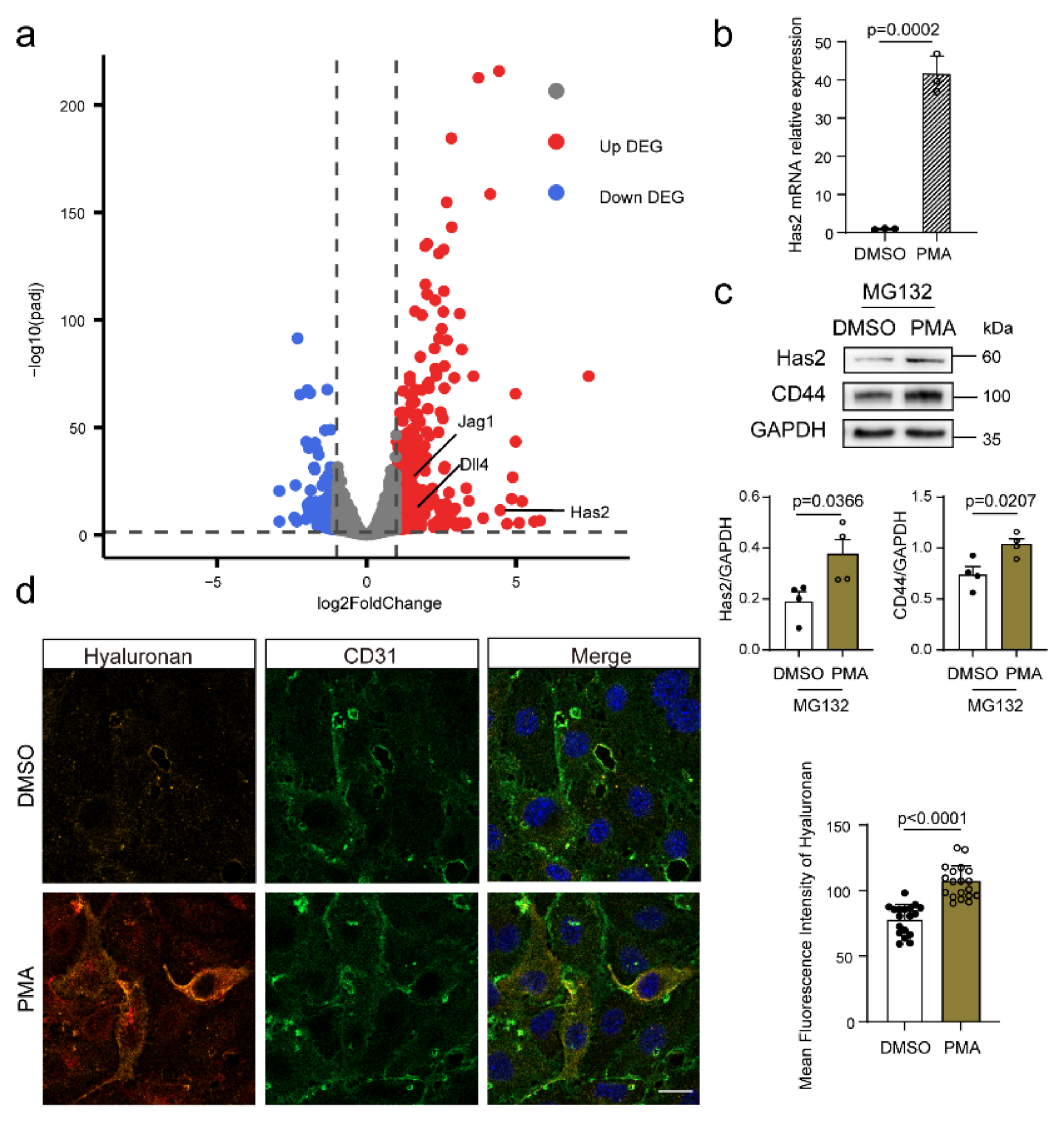
PKC activation upregulates HAS2 expression and promotes hyaluronic acid synthesis. (**a**) Volcano plot of differentially expressed genes in MS1 cells treated with PMA (10 ng/mL, 3 h) versus control. Red and blue dots indicate significantly upregulated and downregulated genes, respectively (|log_2_FC| > 1, adjusted *p* < 0.05). Key genes (Jag1, Dll4, Has2) are labeled. Differential expression analysis was performed using DESeq2. (**b**) RT-qPCR validation of Has2 mRNA in PMA-treated MS1 cells. Expression was normalized to GAPDH and presented as fold change relative to control (2^−ΔΔCt^ method). (**c**) Western blot analysis and quantification of Has2 and CD44 protein levels in MS1 cells treated with PMA (10 ng/mL, 3 h). GAPDH was used as a loading control. (**d**) Immunofluorescence images showing hyaluronic acid (HA, red) distribution in PMA-treated MS1 cells. CD31 (green) marks endothelial cells. Scale bar = 10 μm. Data are presented as mean ± SEM (n = 3 for qPCR; n = 4 for Western blot). Exact *p* values are indicated; statistical analysis was performed using unpaired Student’s *t*-test (**b**–**d**).

To gain a more comprehensive understanding of the impact of PKC activation on Notch signaling in endothelial cells, we performed bulk RNA-seq on PMA-stimulated MS1 cells. As expected, *Jag1* and *Dll4* were significantly upregulated in stimulated cells (Figure 4a). Additionally, we found that *Has2*, the key enzyme responsible for hyaluronic acid synthesis, was also significantly increased (Figure 4a). *Has2* null mice display impaired endocardial–mesenchymal transition in the developing endocardium and develop acellular endocardial cushions [25]. The same phenotype is observed in Notch1-inactivated mouse embryos [26]. This prompted us to hypothesize that PKC may promote Notch activation via stimulation of extracellular hyaluronic acid deposition. We first confirmed *Has2* upregulation at both mRNA and protein levels upon PKC activation. Along with Has2, its receptor protein CD44 was also upregulated (Figure 4b,c). Furthermore, hyaluronic acid content significantly increased as early as 3 h after PKC activation (Figure 4d). These results demonstrate that PKC activation not only promotes Has2 expression but also enhances its functional activity, leading to increased hyaluronic acid synthesis.

### 3.5. Hyaluronic Acid Mediates PKC-Induced Notch Activation

**Figure 5:**
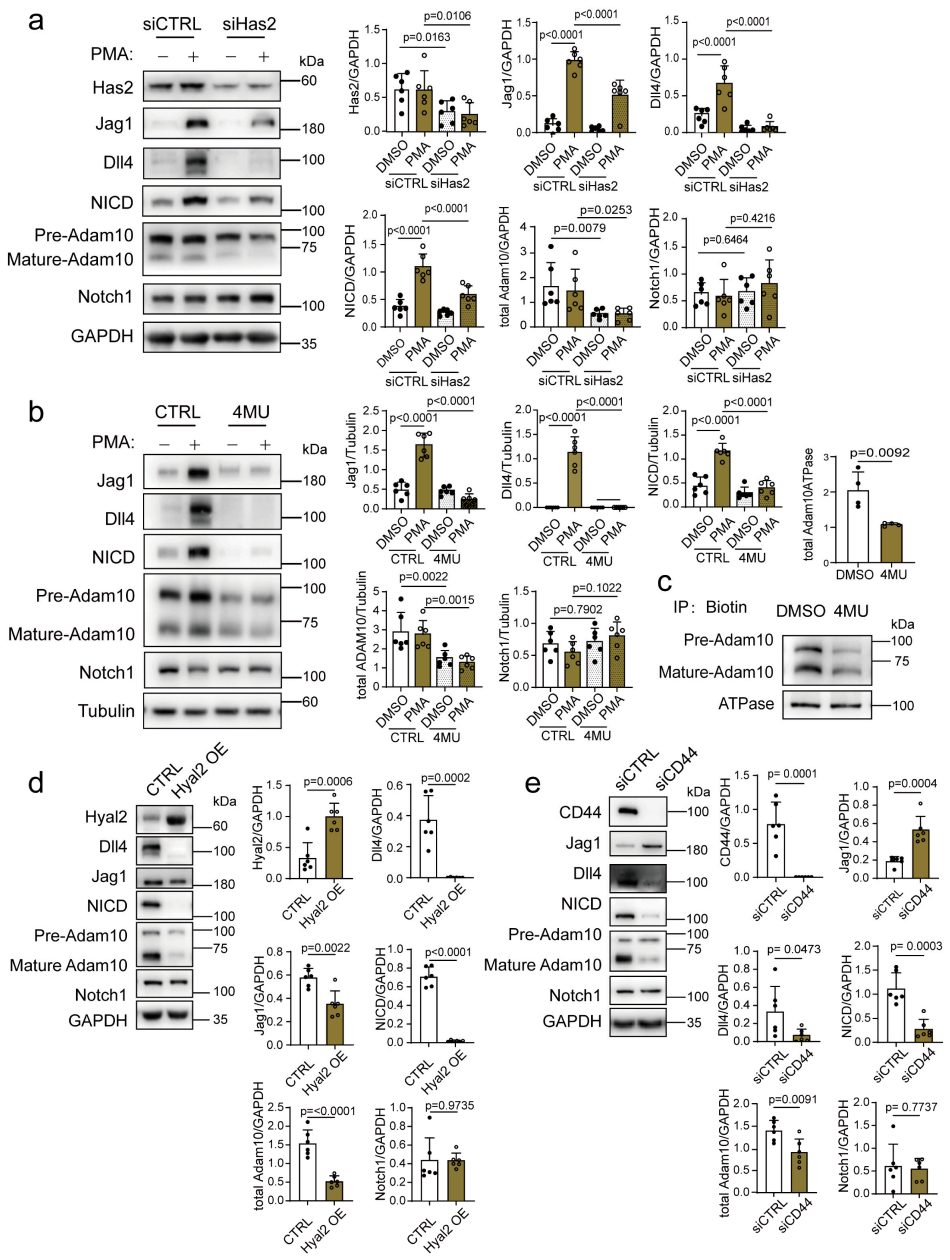
Disruption of the HA-CD44 axis inhibits PKC-mediated NICD generation. (**a**,**b**,**d**,**e**) Western blot analysis and quantification of indicated proteins in MS1 cells treated with PMA (10 ng/mL, 3 h) under the following conditions: (**a**) transfection with HAS2-specific siRNA (siHAS2) or negative control (siNC) for 48 h; (**b**) pretreatment with the hyaluronan synthesis inhibitor 4-methylumbelliferone (4-MU, 1 mM, 12 h); (**d**) overexpression of Hyal2 (Hyaluronidase 2); (**e**) transfection with CD44-specific siRNA (siCD44) or siNC for 48 h. (**c**) Cell-surface biotinylation assay showing surface ADAM10 levels in MS1 cells pretreated with 4-MU (1 mM, 12 h). Na^+^/K^+^-ATPase was used as a membrane fraction loading control. GAPDH and α-Tubulin were used as loading controls for total lysates. Data are presented as mean ± SEM (n = 6 independent experiments for (**a**,**b**,**d**,**e**); n = 4 for (**c**)). Exact *p* values are indicated; statistical analysis was performed using two-way ANOVA with Tukey’s post hoc test (**a**,**b**) or unpaired Student’s *t*-test (**c**–**e**).

To verify whether Has2 is a key molecule involved in PKC-regulated Notch signaling activation, we silenced *Has2* expression and found that Has2 knockdown significantly inhibited NICD activation. *Has2* knockdown did not alter Notch1 protein levels but downregulated the Notch ligands Dll4 and Jag1, with a more pronounced effect on Dll4 expression. Furthermore, Adam10 protein levels also decreased significantly (Figure 5a). We further employed 4-methylumbelliferone (4-MU) to inhibit hyaluronic acid synthesis and found that 4-MU treatment similarly inhibited PMA-induced NICD activation, attenuated the promoting effect of PKC on Jag1 and Dll4, and downregulated Adam10 protein expression, while Notch1 levels remained unchanged (Figure 5b). Consistently, cell-surface biotinylation assays confirmed that 4-MU pretreatment reduced ADAM10 levels at the plasma membrane (Figure 5c), indicating that hyaluronan is required for ADAM10 surface retention. This inhibitory effect of 4-MU on NICD generation was independently confirmed in mouse aortic endothelial cells (MAECs), where 4-MU pretreatment similarly suppressed PMA-induced NICD levels (Figure S4). Overexpression of hyaluronidase 2 (Hyal2) almost completely abolished NICD without affecting Notch1 receptor levels. Adam10, Jag1, and Has2 protein expression was downregulated upon Hyal2 overexpression. Similar to NICD, Dll4 protein was almost completely eliminated, possibly as a downstream effect of Notch inactivation (Figure 5d). Knockdown of CD44, the receptor for hyaluronic acid, also dramatically blocked Notch activation and decreased Adam10 and Dll4 levels (Figure 5e). Surprisingly, Jag1 was increased upon CD44 silencing, suggesting that hyaluronic acid promotes Notch activation also through a ligand-independent pathway, possibly by maintaining an adequate amount of Adam10 protein for Notch cleavage. Collectively, PKC-mediated Notch activation is dependent on hyaluronic acid in addition to its canonical ligands.

## 4. Discussion

**Figure 6:**
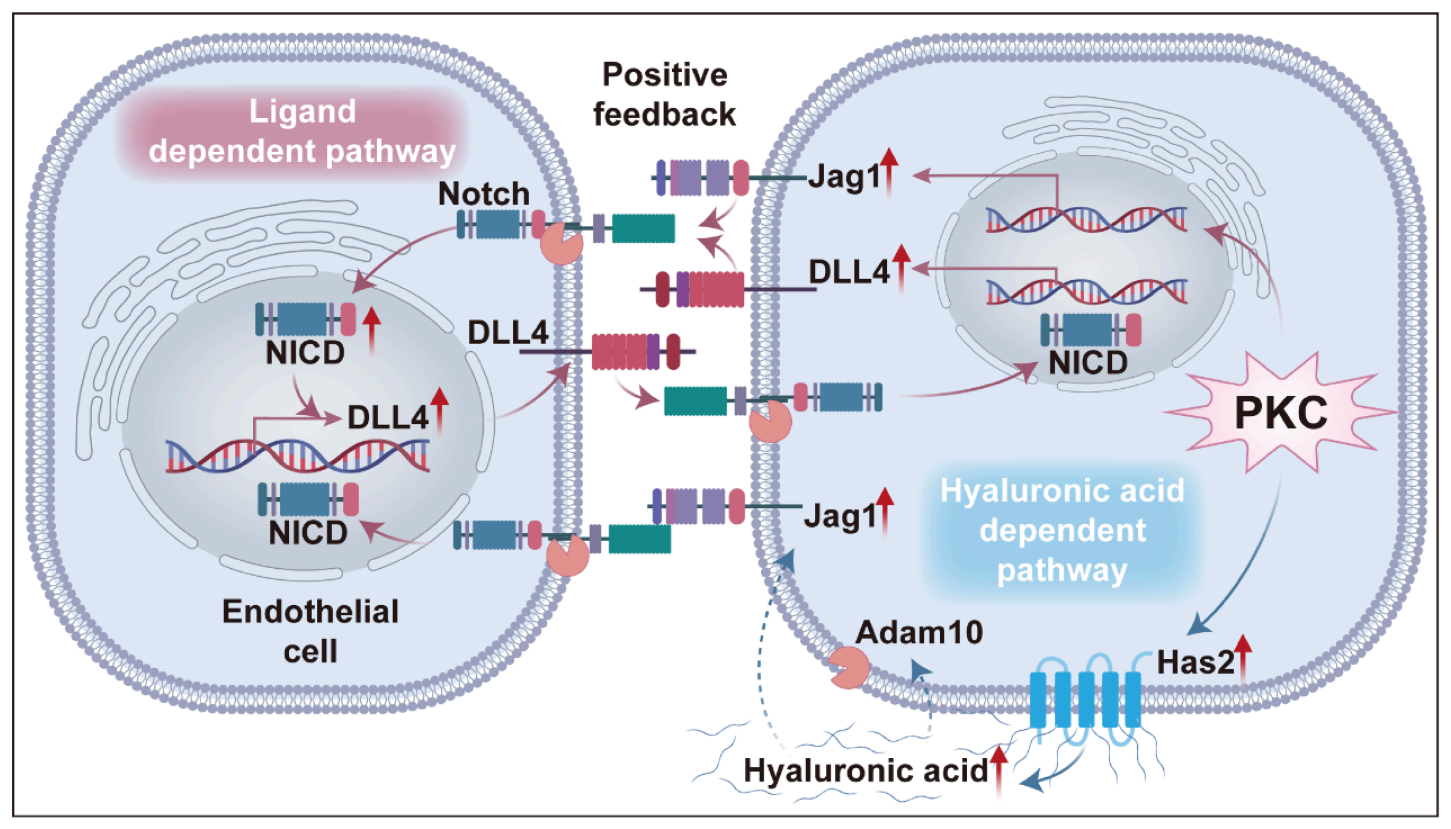
Schematic model of the PKC-induced Notch activation in endothelial cells. PKC activates Notch1 through two parallel mechanisms. First, PKC upregulates Jag1 expression, triggering Notch1 cleavage and NICD release; NICD in turn transactivates Dll4, forming a positive feedback loop. Second, PKC induces Has2-dependent HA synthesis, which maintains ADAM10 protein levels required for Notch1 cleavage via CD44 signaling. Solid arrows, experimentally validated; dashed arrow, proposed mechanism. Arrow colors denote distinct signaling pathways: red, Jag1–Notch1–Dll4 positive feedback loop; blue, Has2–HA–CD44–ADAM10 axis. In this study, we found that PKC upregulates Jag1 expression, which then activates Notch signaling in cultured endothelial cells. A previous study found that PKC activation did not activate Jag1 expression in osteoblasts [31]. Thus this relationship between PKC and Jag1 is cell type and possibly developmental stage-dependent. Regarding Dll4, we observed that its upregulation is blocked by DAPT, indicating that Dll4 is a transcriptional target of Notch signaling itself. This is consistent with previous reports that Dll4 contains RBP-J binding sites in its promoter and is directly transactivated by NICD [32]. In a previous study, double-null mutation of protein kinase C delta and epsilon caused defective blood vessel formation and embryonic lethality at E9.5 [33]. Although the impact of PKC deletion on Notch ligand expression and Notch activity was not examined in that study, it may represent one of the key underlying mechanisms. In our monolayer cell culture system, we cannot assess whether all these signaling components co-exist in the same cells and whether PKC influences lateral inhibition between neighboring cells—specifically, whether Notch receptor expression and Dll4 expression antagonize each other. These need to be investigated in future co-culture systems.

Multiple mechanisms operate to regulate Notch signaling during cell fate determination and developmental pattern formation. These include lateral inhibition of Delta expression and Notch activity during tip/stalk cell specification in sprouting angiogenesis [27] and neuron differentiation [28], asymmetric localization of Numb protein to promote asymmetric cleavage of neural precursor cells [29,30], and VEGF-induced Dll4 expression during arterial fate specification [6,7]. In this study, we identified a novel mechanism in which PKC activity promotes Notch activation through both ligand-dependent and hyaluronic acid-dependent pathways (Figure 6). In this model, PKC induces Jag1 expression, which in turn activates Notch; Dll4, which is transactivated by Notch itself, further stimulates Notch in a positive feedback loop. Additionally, PKC activates Has2 transcription and hyaluronic acid synthesis, and through its receptor CD44, maintains appropriate levels of key Notch signaling components such as Adam10 to mediate Notch cleavage.

PKC has been reported to directly phosphorylate Notch1 at serine residue S1791, which promotes Notch1 processing in late endosomes and enhances its nuclear translocation. To test whether such a direct phosphorylation event contributes to PKC-induced Notch activation in endothelial cells, we mutated the corresponding conserved serine residue S1791 [19]. Unexpectedly, this mutation did not abolish the ability of PKC to activate NICD. This discrepancy suggests that in endothelial cells, PKC may act upstream of Notch through indirect mechanisms. Notably, PKC has also been shown to phosphorylate vimentin, an intermediate filament protein that interacts with Jag1 and is required for proper Notch activation [34]. Vimentin interacts with the spectrin cytoskeleton, which in turn anchors CD44 [35]. Whether PKC might indirectly influence vimentin function through hyaluronic acid and its receptor CD44 remains to be determined.

In addition to its effects on ligand expression, PKC activation strongly induced Has2 transcription and hyaluronic acid synthesis. Importantly, Has2-null mice exhibit a well-characterized phenotype: impaired endocardial–mesenchymal transition (EndMT) and acellular endocardial cushions, leading to embryonic lethality [25]. This phenotype is strikingly similar to that of Notch1-deficient mouse embryos [26]. The source of hyaluronic acid in the developing heart is dual—it is produced by both the endocardium and the myocardium. However, why Has2 deficiency leads to acellular cushions has remained unclear. Our results provide a plausible explanation: loss of Has2 reduces hyaluronic acid availability, which in turn compromises PKC-dependent Notch activation in the endocardium, thereby blocking EndMT and cushion cellularization. Nevertheless, this remains an in vitro observation, and in vivo validation using endothelial-specific Has2 knockout models is warranted. The precise molecular mechanism by which hyaluronic acid is required for Notch activation remains unknown. One possibility is that hyaluronic acid, through its receptor CD44, is necessary for the stabilization and retention of Adam10 on the plasma membrane. In support of this notion, CD44 is a natural substrate of Adam10 and Adam17 [36], although direct evidence linking hyaluronic acid–CD44 signaling to Adam10 stability in endothelial cells is currently lacking. Our observation that CD44 knockdown reduces Adam10 protein levels, together with the finding that Has2 or Hyal2 manipulations also affect Adam10, supports a model in which hyaluronic acid–CD44 signaling maintains an adequate pool of Adam10 at the cell surface to mediate Notch cleavage. However, whether this reduction in ADAM10 reflects decreased protein stability, impaired trafficking to the plasma membrane, or both, was not directly resolved in the current study. Determining ADAM10 subcellular localization—particularly its surface retention—under HA-depleted conditions through live-cell imaging, and measuring its protein half-life via cycloheximide chase assays will be important next steps. Additionally, formal validation of this model will require rescue experiments, such as reintroduction of ADAM10 or exogenous hyaluronic acid under knockdown/inhibitor conditions.

Notch signaling is critically involved in adult vascular homeostasis. In atherosclerotic lesions, disturbed flow alters Notch activity and promotes an inflammatory, pro-atherogenic endothelial phenotype [37]. Notably, RBPJ-mediated epigenetic modifications control macrophage efferocytosis, a central determinant of atherosclerotic lesion progression and resolution. This mechanism directly connects the Notch pathway to plaque biology [38]. Our identification of PKC as a key node linking shear stress to Notch activation suggests that pharmacological modulation of PKC or its downstream effectors (Jag1, hyaluronic acid, ADAM10) might offer therapeutic strategies to restore Notch activity in atheroprone regions. Moreover, in the context of heart valve disease, congenital valve malformations are often linked to aberrant Notch signaling. Understanding how PKC drives Notch activation during valve morphogenesis may inform strategies to promote valve repair or tissue-engineered valve formation.

## 5. Conclusions

We provide evidence to show that PKC activates Notch through dual, parallel mechanisms: (i) transcriptional induction of the Notch ligand Jag1 and (ii) maintenance of ADAM10 protein levels required for Notch proteolytic cleavage. These findings offer a mechanistic explanation for the phenotypic similarity between Has2-null and Notch1-null embryos and reveal new potential therapeutic targets for diseases involving aberrant Notch signaling, including congenital valve malformations and atherosclerosis.

## Data Availability

Further inquiries can be directed to the corresponding author.

## Supplementary Materials

The following supporting information can be downloaded at: https://www.mdpi.com/article/10.3390/cells15151349/s1.
