# Supplementary material for: Protein Kinase C Promotes Notch1 Cleavage Through Both Ligand-Dependent and Hyaluronic Acid–ADAM10-Dependent Mechanisms
Source: Cells. 2026 Jul 27;15(15):1349. doi: 10.3390/cells15151349 (PMC13466031; doi:10.3390/cells15151349)
Supplement: Supplementary file 1 [file cells-15-01349-s001.zip › cells-4318908-supplementary.pdf]

Supplementary Materials

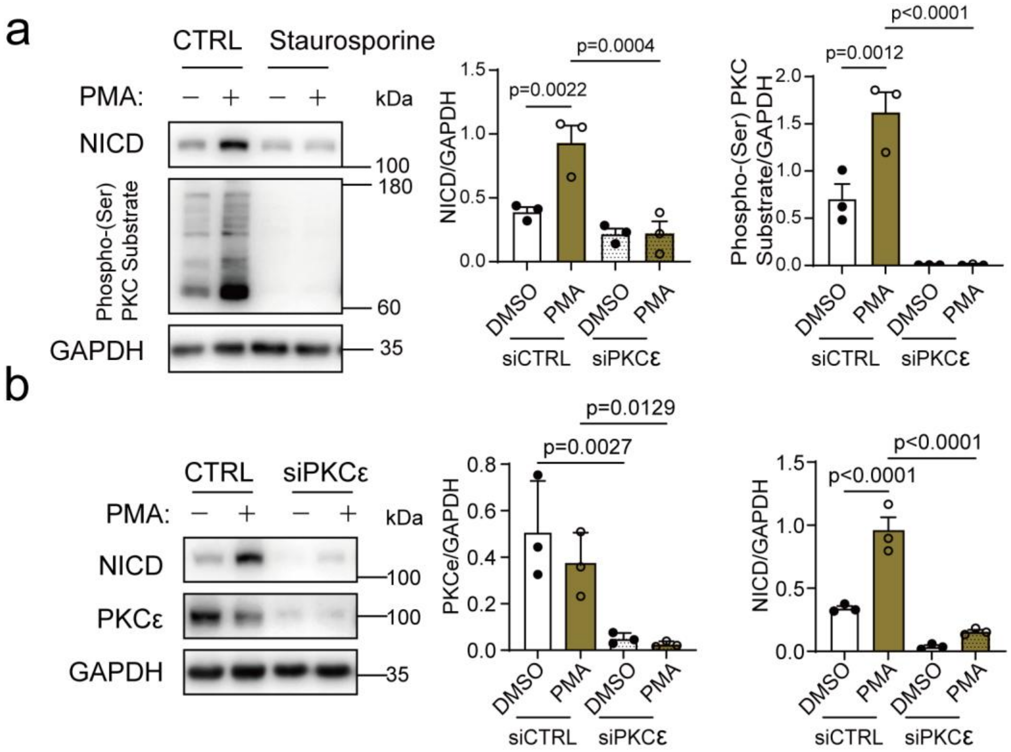

Supplementary Figure S1. PKC-dependent NICD generation in mouse aortic endothelial cells (MAECs). (a,b) Western blot analysis and quantification of indicated proteins in MAECs treated with PMA (10 ng/mL, 3 h) following (a) pretreatment with the PKC inhibitor Staurosporine (1 μM, 3 h) or (b) transfection with PKCε-specific siRNA (siPKCε) or negative control (siNC) for 48 h. GAPDH was used as a loading control. Data are presented as mean ± SEM (n = 3). Exact p values are indicated; statistical analysis was performed using two-way ANOVA with Tukey's post hoc test.

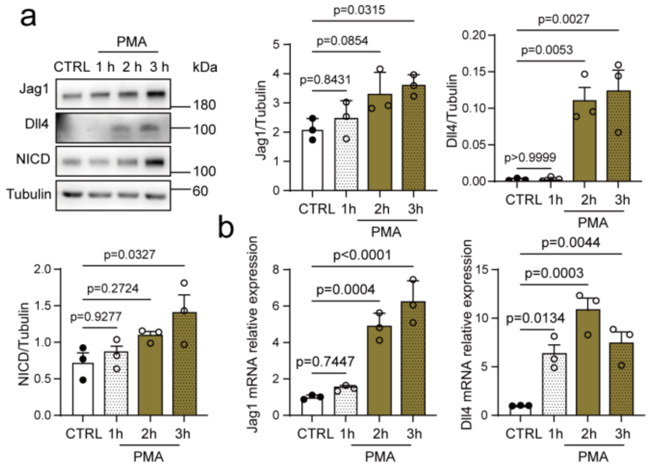

Supplementary Figure S2. PMA-induced PKC activation promotes time-dependent upregulation of Notch1 ligands and NICD generation in MS1 endothelial cells. (a) Western blot analysis and quantification of Jag1, Dll4, and NICD protein levels in MS1 cells treated with PMA (10 ng/mL) for 1, 2, and 3 h. α-Tubulin was used as a loading

control. (b) RT-qPCR analysis of Jag1 and Dll4 mRNA expression at the indicated time points. Expression was normalized to GAPDH (2^−ΔΔCt method) and presented as fold change relative to control. Data are presented as mean ± SEM (n = 3). Exact p values are indicated; statistical analysis was performed using one-way ANOVA with Dunnett's post hoc test.

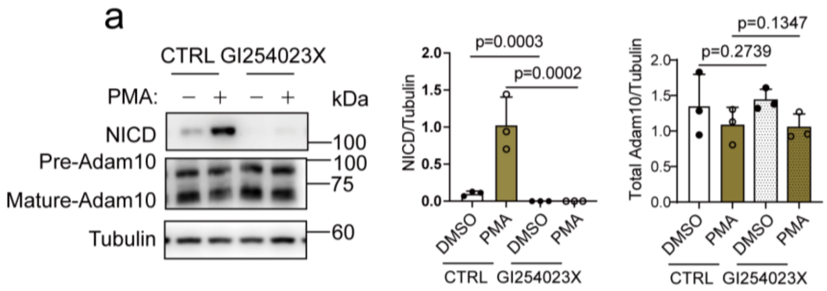

Supplementary Figure S3. ADAM10 protease activity is required for PKC-mediated NICD generation. Western blot analysis and quantification of NICD levels in MS1 cells treated with PMA (10 ng/mL, 3 h) in the presence or absence of the ADAM10-specific inhibitor GI254023X (10 μM). α-Tubulin was used as a loading control. Data are presented as mean ± SEM (n = 3). Exact p values are indicated; statistical analysis was performed using two-way ANOVA with Tukey's post hoc test.

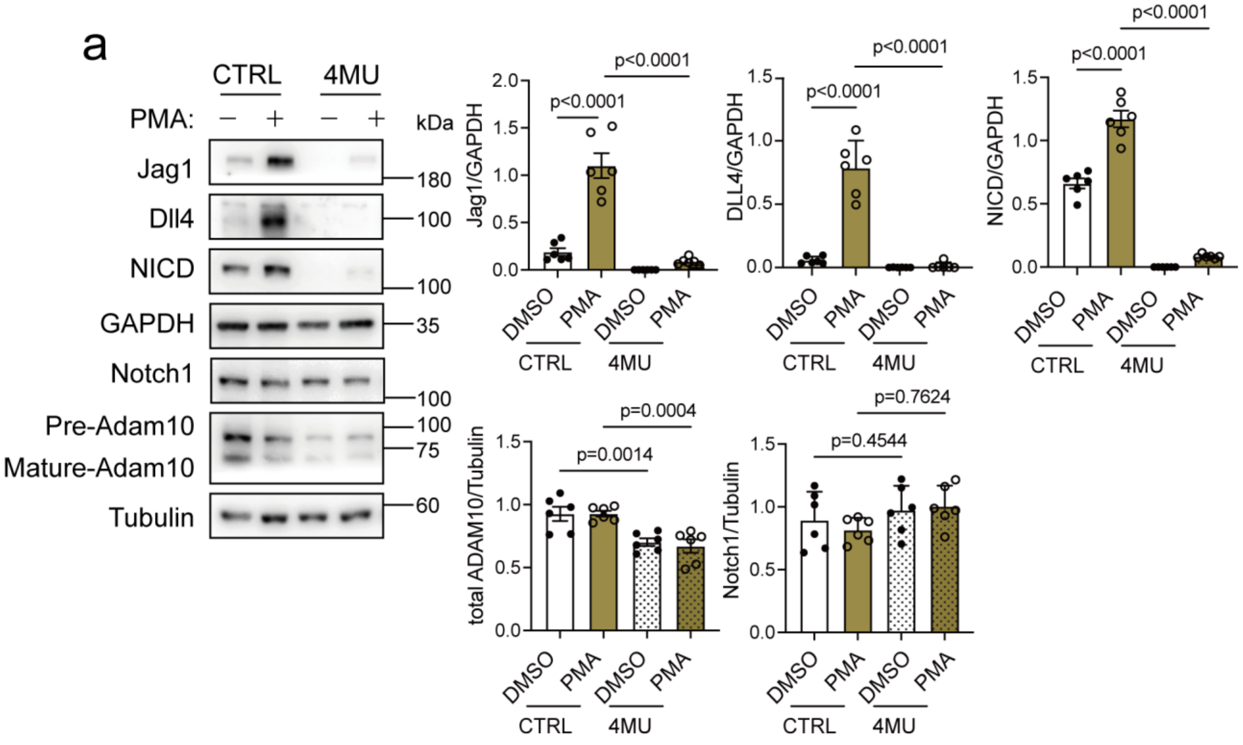

Supplementary Figure S4. Hyaluronan-dependent NICD generation in mouse aortic endothelial cells (MAECs). Western blot analysis and quantification of NICD levels in MAECs pretreated with 4-methylumbelliferone (4-MU, 1 mM, 12 h) followed by PMA

stimulation (10 ng/mL, 3 h). GAPDH and α-Tubulin were used as loading controls. Data are presented as mean ± SEM (n = 6). Exact p values are indicated; statistical analysis was performed using two-way ANOVA with Tukey's post hoc test.
